# Social media, peer norms, and betel nut susceptibility and use: Evidence from early adolescents in Guam

**DOI:** 10.3389/fcomm.2022.960093

**Published:** 2022-07-22

**Authors:** Francis Dalisay, Yoshito Kawabata, Wayne Buente, Pallav Pokhrel, Chantay Benitez, Thaddeus Herzog

**Affiliations:** 1 College of Liberal Arts and Social Sciences, University of Guam, Mangilao, GU, United States; 2 School of Communications, University of Hawai’i at Mānoa, Honolulu, HI, United States; 3 Population Sciences in the Pacific (Cancer Prevention in the Pacific) University of Hawai’i Cancer Center, Honolulu, HI, United States

**Keywords:** betel (areca) nut, social media, peer norms, adolescents, health disparities, adolescent

## Abstract

Betel (areca) nut is an addictive psychoactive substance considered to be carcinogenic. Yet not much is known about factors that may be promoting betel nut use. To fill this void, the present study examines the roles of exposure to betel nut-related posts on social media and peer norms regarding betel nut use in explaining betel nut susceptibility and use among adolescents. We conducted a representative survey of early adolescents (*N* = 673) attending all public middle schools on Guam, a United States-Affiliated Pacific Island in the Western Pacific. Results showed that exposure to betel nut-related posts on social media was positively associated with peer descriptive norms regarding betel nut use. Specifically, the more frequent early adolescents were exposed to betel nut posts on social media, the more likely they were to perceive betel nut use to be more prevalent among their general peers and close friends. Additionally, early adolescents’ exposure to betel nut-related posts on social media was positively associated with both their susceptibility to use betel nut and reported use. Descriptive norms regarding close friends’ betel nut use was also positively associated with both susceptibility to and use of betel nut. However, descriptive norms regarding general peers’ betel nut use was unrelated with either betel nut susceptibility or use. Descriptive norms regarding close friends’ betel nut use mediated the relationships that exposure to betel nut posts on social media had with both betel nut susceptibility and betel nut use. Implications are discussed.

## Introduction

Betel (areca) nut is an addictive psychoactive substance that it is habitually chewed by millions of people around the world (Gupta and Wanakulasuriya, 2002; World Health Organization, 2012). The International Agency for Cancer Research (IACR) classifies betel nut as a Group 1 carcinogen (World Health Organization, 2012). Studies have shown that betel nut use is associated with increased risks of cancers of the head and neck and oral cavity (Gupta, 1991), periodontal disease (Hsiao et al., 2015), cardiovascular disease (Lin et al., 2008), and metabolic syndrome (Yen et al., 2006).

Although evidence indicates that betel nut use initiation occurs during early adolescence (Oakley et al., 2005; Milgrom et al., 2013, 2016), few studies have examined factors that may be associated with susceptibility to and reported use of betel nut among early adolescents. Two important factors that could explain adolescents’ betel nut use are exposure to betel nut-related posts on social media and peer norms. Social media are ubiquitous in the lives of adolescents (Pew Research Center, 2018). Substance-related posts on socialmedia have the potential to shape behaviors through the influence of peer norms (Nesi et al., 2017). Indeed, a robust paradigm of research has also established peer norms as a strong correlate of adolescents’ substance use (Borsari and Carey, 2001; Hoffman et al., 2006). Two recent studies suggest peer norms could be encouraging betel nut use among adolescents (Dalisay et al., 2019; Pokhrel et al., 2019).

The present study looks into the potential roles of social media and peer norms in promoting early adolescents’ susceptibility to, and use of betel nut. We specifically focus on peer descriptive norms, or beliefs regarding how prevalent a particular behavior is among one’s peers (Borsari and Carey, 2001). Building on prior research that has revealed associations between exposure to substance-related messages on social media, peer descriptive norms, and substance use (e.g., Beullens and Vandenbosch, 2016; Nan and Zhao, 2016), we seek to accomplish four goals. First, we examine whether early adolescents’ exposure to betel nut posts on social media is positively associated with their susceptibility to and reported use of betel nut. Second, we examine whether exposure to betel nut posts on social media is positively associated with peer descriptive norms regarding the use of betel nut. Third, we analyze whether peer descriptive norms regarding betel nut use are positively associated with betel nut susceptibility and use. Fourth, we test whether exposure to betel nut posts on social media will have an indirect positive association with both betel nut susceptibility and use *via* peer descriptive norms.

We conduct this study on Guam, a United States-Affiliated Pacific Island (USAPI) in the Western Pacific. A World Health Organization (2012) report noted that betel nut chewing is an urgent public health threat faced by the Western Pacific region. Approximately 50% of adults in the USAPI chew betel nuts at least once a week (World Health Organization, 2012). These high rates of betel nut chewing correspond with the high rates of cancer in the USAPI (Gupta, 1991; Paulino et al., 2011). Oral and lung cancer incidences and mortality rates are higher in the USAPI region than on the mainland U.S., and such health disparities could be linked in part to betel nut chewing (Paulino et al., 2011). Research shows that the average age of betel nut use initiation among USAPI adolescents is ~11 years (Milgrom et al., 2016). Among early adolescents in Guam (average age ~12 years), an estimated 8% report having used betel nut within the past month (Dalisay et al., 2019).

Our study tests the following hypotheses:

H1: Exposure to betel nut-related posts on social media will be positively associated with peer descriptive norms regarding betel nut use.

H2: Exposure to betel nut-related posts on social media will be positively associated with (a) susceptibility to use betel nut and (c) betel nut use.

H3: Peer descriptive norms regarding betel nut use will be positively associated with (a) susceptibility to use betel nut and (b) betel nut use.

H4a: Exposure to betel nut-related posts on social media will have an indirect positive association with susceptibility to use betel nut through peer descriptive norms regarding betel nut use.

H4b: Exposure to betel nut-related posts on social media will have an indirect positive association with betel nut use through peer descriptive norms regarding betel nut use.

## Methods

We administered the survey in 2017 to 2018.^1^ The survey was part of a larger study on substance use among adolescents in Guam. The regional Institutional Review Board (IRB) committee approved the study. Permission to conduct the study was also granted by the Guam Department of Education (GDOE) and the principals of each of the 8 public middle schools.

We randomly selected 4 classes per school, and research staff collected data in the classrooms. The average class size per school was ~20–30 students. The total sampling frame comprised of 882 students. Both parental consent and student assent were sought before students participated in the survey. Participants were assured that their responses would remain anonymous as the survey did not ask for any identifying information. Surveys were self-administered and facilitated by a PI and research assistant. The survey contained 73 items and took ~30 min to complete. As compensation for their time and effort, all students, regardless of whether or not they chose to participate in the survey, received a $5 gift certificate to a local chain cinema and a free ballpoint pen.

After excluding surveys that were <75% complete, we obtained a total of *N* = 673 completed surveys.^2^ The overall response rate was 76%. The ages of participants ranged from 10 to 14 years (with the mean age around 12.5 years), with 51% male (*n* = 343) and 49% female (*n* = 330), which by comparison, are relatively close to the age and gender numbers of the true population of Guam’s public middle school students. Other characteristics of the sample are provided in Table 1 and briefly reported below.

## Measures

### Independent variable

#### Exposure to betel nut-related posts on social media

Exposure to posts relevant to betel nut on social media was measured by 5 items. The 5 items asked respondents how often they see posts related to betel nut on the following social media platforms: (a) Facebook, (b) Twitter, (c) Instagram, (d) Snapchat, and (e) WhatsApp (0 = I don’t use this, 1 = never, 2 = rarely, 3 = sometimes, 4 = often).^3^ We merged responses for “I don’t use this” and “never” under a single category so that the scale had 4 points (1 = I don’t use this/never, 4 = often). Responses to the 5 items were combined and averaged to form a single construct (*M* = 1.28, *SD* = 0.51, α = 0.88).

### Mediating variables

#### Peer descriptive norms regarding betel nut use

We employed two measures for descriptive peer norms. The items were adapted from previous adolescent substance use research (Flay et al., 1998; Janssen et al., 2018). The first was a measure assessing peer descriptive norms regarding betel nut use among general peers. This measure comprised of the following two items: (a) out of every 100 male students your age at your school, how many do you think chew betel-nut at least once a week, and (b) out of every 100 female students your age at your school, how many do you think chew betel nut at least once a week. Responses were measured along a 10-point scale (1 = 10 or less, 2 = 11–20, 3 = 21–30, 4 = 31–40, 5 = 41–50, 6 = 51–60, 7 = 61–70, 8 = 71–80, 9 = 81–90, 10 = 91–100). The two items, which were highly correlated (*r* = 0.75, *p* < 0.001), were combined and averaged to form a single index (*M* = 2.29, *SD* = 2.02). The second measure assessed descriptive norms regarding betel nut use among close friends, using the following single item: how many of your five closest friends usually chew betel nut at least once a week. Responses were measured along a 6-point scale, where 0 = no friends, 1 = 1 friend, 2 = 2 friends, 3 = 3 friends, 4 = 4 friends, and 5 = 5 friends (*M* = 0.44, *SD* = 1.19). A higher score indicated a more positive perception of peer descriptive norms.

### Dependent variables

#### Susceptibility to use betel nut

Susceptibility to use betel nut and betel nut use were measured with items adapted from a widely used surveillance measure for adolescent smoking developed by Pierce et al. (1995). Four items measured betel nut use susceptibility: (a) do you think you will use a betel nut soon, (b) do you think you will use a betel nut in the next year, (c) do you think that in the future you might experiment with using a betel nut, and (d) if one of your best friends were to offer you a betel nut, would you use it. Responses were measured along a 5-point Likert scale (1 = definitely not, 5 = definitely yes). We combined and averaged responses to form a single index (*M* = 1.45, *SD* = 0.84, α = 0.89).

#### Betel nut use

We measured betel nut use by asking participants: in the past 30 days (i.e., 1 month), on how many days did you chew a betel nut or a betel quid. Responses were measured along a 7-point Likert scale (1 = 0 days, 2 = 1–2 days, 3 = 3–5 days, 4 = 6–9 days, 5 = 10–19 days, 6 = 20–29 days, 7 = chewed daily) (*M* = 1.14, *SD* = 0.61).

### Covariates

We measured several demographic variables as covariates. The demographic variables included age (measured along the following scale: 1 = 10 years old, 2 = 11 years old, 3 = 12 years old, 4 = 13 years old, 5 = 14 years old, 6 = 15 years old; *M* = 3.66, *SD* = 0.89), sex (1 = male, 2 = female; *M* = 1.49, *SD* = 0.50), grade (1 = 6th grade, 2 = 7th grade, 3 = 8th grade; *M* = 2.06, *SD* = 0.79), ethnicity (Chamorro, Filipino, from the Freely Associated States, and other—including other Asian, Caucasian, Pacific Islander, etc.),^4^ household crowdedness (measured by the number of rooms reported in the participants’ home divided by the number of living in the home; *M* = 0.69, *SD* = 0.39), which serves as a proxy for socioeconomic status (e.g., Centerwall, 1984), and impulsivity (*M* = 2.76, *SD* = 0.87), a known predictor of substance use initiation and risk (Gullo and Dawe, 2008); participants’ reports of whether or not (0 = no, 1 = yes) their fathers, mothers, siblings, and other relatives used betel nut.

### Statistical analyses

A series of OLS regression models (Cohen et al., 2002) were employed to analyze the data and test the proposed hypotheses. We employed Hayes’ (2022) method to test for mediation. These models and the OLS regression results are shown on Tables 4, 5. These models tested the proposed relationships between exposure to betel nut posts on social media and susceptibility to use betel nut (Models 3 and 4 on Table 4) and use of betel nut (Models 3 and 4 on Table 5) (H1). These models also tested the proposed association between exposure to betel nut posts on social media and descriptive peer norms (Models 1 and 2 of Tables 4, 5) (H2). An additional model examined the relationship between descriptive peer norms and betel nut susceptibility and use (Model 4 on Tables 4, 5) (H3). Finally, the SPSS PROCESS macro (Hayes, 2022) was used to estimate the indirect effect of the individual-level indicators of exposure to betel nut posts on social media on susceptibility to use and use through the mediating variable of descriptive peer norms (H4). To infer indirect effects, we used bootstrap methods with 5,000 bootstrap samples and 95% bias-corrected confidence intervals. Listwise deletion was used to deal with missing data.

## Results

### Descriptive statistics

Roughly 7% of the early adolescent participants reported using betel nut in the past 30 days. Tables 1-3 report other relevant descriptive statistics. As shown on Table 1, the majority of the participants identified as Chamorro (47.8%), followed by those originating from the Freely Associated States (27.2%), and Filipino (23%). As shown on Table 2, participants perceived that more than half (51%) of males in their schools chewed betel nut, while less than half (36%) of females were perceived to use betel nut. Roughly 5% of participants reported having one close friend who chewed betel nut once a week; about 4% reported having 2 close friends who chewed each week; about 4% reported having 5 close friends who used betel once a week. Also, about 15% of participants reported that their fathers used betel nut, while roughly 9% reported their mothers used, around 7% reported their siblings used, and around 37% reported that other relatives used.

Results on Table 3 indicate that participants reported being exposed to betel nut posts most frequently on Facebook and Instagram, with over 20% of participants reporting being exposed to betel nut posts on these two social media platforms. Table 3 also shows the frequencies in which participants reported that they do not use each of the respective platforms. These results suggest that among the five platforms, Instagram was used most frequently, followed in descending order by Snapchat, Facebook, Twitter, and WhatsApp. In addition, we further analyzed the same data reported on Table 3 to examine cross-platform usage. We found that among our participants, 35.2% reported using all 5 of the social media platforms, 12.7% reported using 4, 18.8% reported using 3, 10.9% reported using 2, and 9.9% reported using 1. One the other hand, 12.6% reported they did not use any of the 5 platforms. These results indicate that around 87% of the participants reported using 2 or more social media platforms. This may have increased the likelihood of cross-platform exposure to betel nut-relevant posts.

H1 tested whether exposure to betel nut-related posts on social media will be positively associated with peer descriptive norms. Results show support for this hypothesis. As shown on Models 1 and 2 of Tables 4, 5, exposure to betel nut posts on social media is positively related with both descriptive norms, or the perceived prevalence of betel nut use, for general peers (Table 4: β = 0.169, *p* < 0.001; Table 5: β = 0.173, *p* < 0.001) and close friends (Table 4: β = 0.229, *p* < 0.001; Table 5: β = 0.232, *p* < 0.001).

H2 proposed that exposure to betel nut-related posts on social media will be positively associated with (a) susceptibility to use betel nut and (b) use. The OLS regression results shown on Model 3 of Tables 4, 5 show that exposure to betel nut posts on social media is positively associated with both betel nut susceptibility (β = 0.133, *p* < 0.001) and use (β = 0.167, *p* < 0.001). As shown on Table 4, when peer descriptive norms are controlled as predictors in Model 4, the positive link between exposure to betel nut posts on social media and betel nut susceptibility remains significant (β = 0.092, *p* <0.05). Similarly, as Model 4 on Table 5 shows, the positive relationship between exposure to betel nut posts on social media and betel nut use remains significant (β = 0.109, *p* < 0.01).

H3 predicted that peer descriptive norms are positively associated with (a) susceptibility to use and (b) use of betel nut. The OLS regression results shown on Model 4 of Table 4 indicates that the perceived prevalence of betel nut use among close friends (β = 0.134, *p* < 0.01) is positively associated with betel nut susceptibility. However, the relationship between descriptive norms of general peers and betel nut susceptibility was not statistically significant (β = 0.060, *p* < 0.10). Similarly, the Model 4 on Table 5 shows that descriptive norms of close friends’ betel nut use (β = 0.226, *p* < 0.001) was positively associated with one’s self-reported betel nut use. Yet perceived descriptive norms of general peers’ betel nut use were not associated with one’s betel nut use (β = 0.029, *p* < 0.10). Results show partial support for H3, suggesting that the perceived prevalence of betel nut use among one’s close friends— i.e., descriptive norms regarding close friends’ use—is more important than the perceived prevalence of betel nut use among general peers—i.e., norms of general friends—when it comes to predicting early adolescents’ betel nut susceptibility and use.

H4a proposed that exposure to betel nut-related posts on social media will be indirectly positively associated with susceptibility to use betel nut through peer descriptive norms. Results on Table 6 indicate that exposure to betel nut posts on social media did not have an indirect relationship with susceptibility to use *via* descriptive norms among general peers [point estimate = 0.010 (SE = 0.009), 95% CI: −0.004 to 0.031]. However, exposure to betel nut posts on social media had an indirect association with susceptibility through descriptive norms (or perceived prevalence of use) for close friends [point estimate = 0.031 (SE = 0.014), 95% CI: 0.007–0.061]. A similar set of findings emerged for tests of H4b. That is, exposure to betel nut posts on social media was indirectly linked with betel nut use through descriptive norms regarding close friends’ betel nut use [point estimate = 0.052 (SE = 0.023), 95% CI: 0.015–0.105]. However, being exposed to betel nut posts on social media was not indirectly related with reported betel nut use through descriptive norms regarding general peers’ betel nut use [point estimate = 0.005 (SE = 0.010), CI: −0.015 to 0.026]. Taken together, the results show partial support for H4.

## Discussion

Prior research has shown that exposure to substance-related posts on social media (e.g., Depue et al., 2015; Yoo et al., 2016) and peer descriptive norms (e.g., Hoffman et al., 2007; Beullens and Vandenbosch, 2016; Nan and Zhao, 2016) are positively related with substance use susceptibility and use. The present study’s findings contribute to the current literature in several ways. First, our findings indicate that adolescents who are exposed to betel nut-related posts on social media may view the use of betel nut as relatively more prevalent and may believe that using betel nut is relatively more acceptable, as compared to their peers and friends who are less exposed to betel-related posts.

Second, our study showed that adolescents’ exposure to betel nut posts on social media is positively associated with both susceptibility to use betel nut and betel nut use. The associations between exposure to betel nut posts on social media and betel nut use susceptibility, on the one hand, and betel nut use, on the other, remained statistically significant even after accounting for perceived descriptive peer norms. These findings reinforce previous research showing that exposure to substance use-related content on social media could promote substance use (e.g., Depue et al., 2015; Pokhrel et al., 2018).

Third, our results revealed that perceived descriptive norms for general peers (or the perceived prevalence of betel nut use among general peers) were not associated with either betel nut susceptibility or use. Yet descriptive norms for close friends (the perceived prevalence of betel nut use among one’s close friends) were predictive of both betel nut susceptibility and use. As we noted above, relative to the behaviors of general peers, friends’ behaviors may more strongly influence the ways by which adolescents decide to use substances (e.g., Hoffman et al., 2007), such as betel nut, due to the greater levels of emotional closeness and intimacy of friendships. This view is in line with the finding of Janssen, Cox, Merrill, Barnett, Sargent and Jackson (2018) study, which showed that close friend norms were a stronger predictor for youth’s alcohol use than general peer norms. Yet from a cultural perspective, since Guam’s culture is predominantly collectivistic (Dalisay, 2012), it could be that youth on the island may be more susceptible to norms among their close friends (due to the significance of peer pressure for adolescents and cultural norms) rather than the mere perceived prevalence of betel nut use of general peers within their schools. Given that youth are emotionally closer and more susceptible to their close friends than general peers, such as other students in their schools (e.g., Hoffman et al., 2007), close friends may have a stronger influence on the way by which adolescents perceive and determine the use of betel nut.

Our study showed that perceived descriptive norms of betel nut use among close friends served as mediators for the link between exposure to betel nut-related social media posts and reported betel nut use. Similarly, Janssen et al. (2018) found that exposure to social media that portray alcohol use was predictive of youth’s alcohol use *via* close friend descriptive norms, reflecting the perception of alcohol use. On the other hand, our study showed that descriptive norms for betel nut use among general peers did not mediate the links between exposure to betel nut posts on social media and intentions to use and reported use. One explanation is that adolescents may be more likely exposed to posts from their close friends rather than general peers. Indeed, previous research suggests adolescents’ networks on social media are more likely to comprise of close friends rather than general acquaintances (Mazur and Richards, 2011), and they engage more with close friends rather than general acquaintances on social media (Rousseau et al., 2019).

Our study generated promising results; however, several limitations should be acknowledged. First, given that the target populations of the study were adolescents in Guam, the findings could not be generalized and applied to other age and cultural groups. Second, the cross-sectional and correlational nature of the study prevented us from concluding the causality of the associations between exposure to betel nut-related posts on social media, descriptive peer norms, and betel nut susceptibility and use. Although this study revealed that social media predicted betel nut use *via* peer norms, there is a plausibility of a bidirectional effect. In other words, social media may predict betel nut use, but it is also plausible that betel nut use may increase youth’s interest in social media posts related to betel nut. In addition, perceived descriptive norms for general peers and close friends may predict betel nut use, but it is also plausible that betel nut use may increase peer descriptive norms, which in turn, may lead to greater interest in betel nut content on social media. Relatedly, the descriptive results we reported suggest that some participants might not have used one or more of social media investigated in the present study. If a participant did not use a given platform, he or she could not have been exposed to betel content on the platform. However, because we found that a majority of the participants reported using multiple platforms, this could have increased the likelihood of exposure to betel nut content. Additionally, a recent meta-analysis on adolescent social media use and risky behavior determined that visual social media (i.e., Instagram or Snapchat) had greater effects on substance use than other social media platforms (Vannucci et al., 2020). Therefore, it is not only exposure through the number of social media platforms, but also the type of social media platform that may significantly influence perceptions and behaviors. Nevertheless, given the cross-sectional nature of the present study, future research employing experimental and/or longitudinal approaches should be conducted to examine the processes involving social media, norms, peer- and friend-linked factors, and betel nut use. Finally, research has suggested that injunctive norms also may play a role in influencing risk factors for substance use (Lac and Donaldson, 2021), however, our survey did not include any direct measures for injunctive norms. Therefore, we urge future research to look at the potential role of injunctive norms in influencing betel nut use.

Overall, the present study suggests that betel nut-linked posts on social media is a significant risk factor for intentions to use and actual use of betel nut; however, peer and close friend norms partially may explain why this is the case. In other words, social media seem to be impacting use and intentions to use betel nut among adolescents by way of reinforcing perceived norms. It is conceivable that social media and peer- and friend- norms are risk or protective factors, depending on the nature and content of these factors. In effect, campaigns aimed at preventing betel nut use may employ social media platforms to disseminate prevention-related messaging.

## Figures and Tables

**TABLE 1:** Participant characteristics.

	Frequency (%)
**Age**	
11	57 (8.5%)
12	242 (35.9%)
13	252 (37.4%)
14	118 (17.5%)
**Grade**	
6th	189 (28%)
7th	256 (37.9%)
8th	230 (34.1%)
**Sex**	
Female	343 (51%)
Male	330 (49%)
**Ethnicity**	
Chamorro	320 (47.8%)
Filipino	154 (23.0%)
Freely associated states	182 (27.2%)
Other	13 (1.9%)
**Reported betel nut use among family**	
Father use	99 (14.7%)
Mother use	60 (8.9%)
Siblings use	48 (7.1%)
Other relatives use	250 (37.2%)

**TABLE 2:** Descriptive peer norms.

	Frequency (%)
**Male students out of every 100 who chew betel nut at least once a week**	
10 or less	321 (49%)
11–20	121 (18.5%)
21–30	68 (10.4%)
31–40	36 (5.5%)
41–50	37 (5.6%)
51–60	19 (2.9%)
61–70	8 (1.2%)
71–80	4 (0.6%)
81–90	8 (1.2%)
91–100	33 (5.5%)
**Female students out of every 100 who chew betel nut at least once a week**	
10 or less	419 (64%)
11–20	107 (16.3%)
21–30	46 (7.0%)
31–40	30 (4.6%)
41–50	13 (2.0%)
51–60	13 (2.0%)
61–70	3 (0.5%)
71–80	6 (0.9%)
81–90	7 (1.1%)
91–100	11 (1.7%)
**Number of 5 closest friends who chew betel nut at least once a week**	
None	560 (83.8%)
1	36 (5.4%)
2	24 (3.6%)
3	11 (1.6%)
4	9 (1.3%)
5	28 (4.2%)

Perceived number of general peers and close friends who use betel nut.

**TABLE 3:** Frequency of exposure to betel nut posts on social media.

	Exposure to betel nut posts on social media frequency (%)				
	Don’t use	Never	Rarely	Sometimes	Often
Facebook	284 (45.2%)	202 (32.2%)	57 (9.1%)	54 (8.6%)	31 (4.9%)
Twitter	342 (53.9%)	238 (37.5%)	22 (3.5%)	16 (2.5%)	16 (2.5%)
Instagram	141 (22.0%)	371 (57.8%)	67 (10.4%)	32 (5.0%)	31 (4.8%)
Snapchat	195 (31.0%)	356 (56.6%)	30 (4.8%)	27 (4.3%)	21 (3.3%)
WhatsApp	177 (27.8%)	381 (59.9%)	34 (5.3%)	19 (3.0%)	25 (4.0%)

**TABLE 4:** Predictors of susceptibility to use betel nut.

	Peer descriptive norms regarding betel nut use—general peers	Peer descriptive norms regarding betel nut use—close friends	Susceptibility to use betel nut	
	Model 1	Model 2	Model 3	Model 4
Age	−0.021	−0.018	0.090	0.090
Sex (female)	−0.031	0.033	−0.044	−0.046
Grade	−0.002	−0.001	−0.118^#^	−0.118^#^
Ethnicity-Filipino	0.098	−0.028	−0.055	−0.057*
Ethnicity-from the freely associated states	−0.001	0.178***	0.163***	0.139**
Ethnicity-other	−0.032	−0.016	−0.077*	−0.073*
SES-Household crowdedness	0.063	−0.018	0.076*	0.075*
Impulsivity	0.050	0.070^#^	0.135***	0.122**
Father betel nut use	−0.009	−0.005	0.106**	0.107**
Mother betel nut use	0.011	0.105*	0.007	−0.008
Siblings betel nut use	0.029	0.076*	0.083*	0.071^#^
Other relatives betel nut use	0.101*	0.017	0.078*	0.070^#^
Exposure to betel nut-related posts on social media	0.169***	0.229***	0.133***	0.092*
Peer descriptive norms regarding betel nut use—general peers	-	-	-	0.060
Peer descriptive norms regarding betel nut use—close friends	-	-	-	0.134**
***R*^2^ (%)**	**4.67%**	**17.17%**	**17.94%**	**20.11%**

Cell entries are standardized regression coefficients. N = 622.

*** p < 0.001

** p < 0.01

* p < 0.05

# <0.10.

**TABLE 5:** Predictors of betel nut use.

	Peer descriptive norms regarding betel nut use—general peers	Peer descriptive norms regarding betel nut use—close friends	Betel nut use	
	Model 1	Model 2	Model 3	Model 4
Age	−0.013	−0.007	0.111	0.113^#^
Sex (female)	−0.037	0.032	−0.064	−0.070^#^
Grade	−0.018	−0.017	−0.049	−0.045
Ethnicity-Filipino	0.100*	−0.029	−0.069*	−0.066
Ethnicity-from the freely associated states	0.010	0.175***	0.036	0.003
Ethnicity-other	−0.032	−0.013	−0.023	−0.019
SES-Household crowdedness	0.063	−0.026	0.028	0.032
Impulsivity	0.049	0.071^#^	0.055	0.038
Father betel nut use	0.004	0.006	0.101*	0.099*
Mother betel nut use	−0.008	0.093*	0.027	0.006
Siblings betel nut use	0.032	0.079*	0.004	−0.015
Other relatives betel nut use	0.100*	0.011	0.019	0.014
Exposure to betel nut-related posts on social media	0.173***	0.232***	0.167***	0.109**
Peer descriptive norms regarding betel nut use—general peers	-	-	-	0.029
Peer descriptive norms regarding betel nut use—close friends	-	-	-	0.226***
***R*^2^ (%)**	**4.80%**	**17.11%**	**8.87%**	**13.45%**

Cell entries are standardized regression coefficients. N = 611.

*** p < 0.001

** p < 0.01

* p < 0.05

# <0.10.

**TABLE 6:** Indirect associations between exposure to betel nut-related posts on social media and betel nut susceptibility and use through perceived descriptive peer norms.

Indirect associations	Effect
Exposure to betel nut-related posts on social media → Peer descriptive norms regarding betel nut use—general peers → Susceptibility to use betel nut	0.010 (SE = 0.009), 95% CIs (−0.004, 0.031)
Exposure to betel nut-related posts on social media → Peer descriptive norms regarding betel nut use—close friends → Susceptibility to use betel nut	0.031 (SE = 0.014), 95% CIs (0.007, 0.061)
Exposure to betel nut posts on social media → Peer descriptive norms regarding betel nut use—general peers → Betel nut use	0.005 (SE = 0.010), 95% CIs (−0.015, 0.026)
Exposure to betel nut posts on social media → Peer descriptive norms regarding betel nut use—close friends → Betel nut use	0.052, (SE = 0.023), 95% CIs (0.015, 0.105)

## Data Availability

The datasets presented in this article are not readily available because of restrictions from the governing IRB that reviewed and approved the study. Requests to access the datasets should be directed to fdalisay@triton.uog.edu.

## Footnotes

1 Strategies from Dillman et al.’s (2014) Tailored Design Method were used to execute the survey, and in particular, to design the layout of the survey. In addition, prior to collecting data in the schools, the research team conducted cognitive interviews with 5 adolescents in to identify any issues related to measurement error (e.g., poor wording of question items and fatigue). The results of the cognitive interviews were adapted to improve the design of the survey instrument.

2 Assuming a two-tailed Bonferroni adjusted Type I error rate of 0.01, a small-to-medium effect size (*r* = 0.10–0.25) for the effect of the independent variable on substance use and susceptibility and an R-square for covariates equal to 0.40, a sample size of *N* = 600 was found to ensure over 80% power.

3 These 5 social media platforms were selected based on in-depth interviews of over 20 adolescents on Guam conducted in 2016. The adolescents interviewed reported the above 5 social media platforms as the ones they used most frequently on a daily basis.

4 We treated Chamorros as the reference group and omitted this group as an ethnicity predictor in the regression models. Thus, we included only Filipinos, those originating from the Freely Associated States, and all other ethnicities as covariates in our regression models.
